# Fumarate Production by *Torulopsis glabrata*: Engineering Heterologous Fumarase Expression and Improving Acid Tolerance

**DOI:** 10.1371/journal.pone.0164141

**Published:** 2016-10-06

**Authors:** Xiulai Chen, Wei Song, Cong Gao, Wen Qin, Qiuling Luo, Jia Liu, Liming Liu

**Affiliations:** 1 State Key Laboratory of Food Science and Technology, Jiangnan University, Wuxi, China; 2 Key Laboratory of Industrial Biotechnology, Ministry of Education, Jiangnan University, Wuxi, China; 3 Laboratory of Food Microbial-Manufacturing Engineering, Jiangnan University, Wuxi, China; Leibniz-Institut fur Pflanzengenetik und Kulturpflanzenforschung Gatersleben, GERMANY

## Abstract

Fumarate is a well-known biomass building block compound. However, the poor catalytic efficiency of fumarase is one of the major factors preventing its widespread production. To address this issue, we selected residues ^159^HPND^162^ of fumarase from *Rhizopus oryzae* as targets for site-directed mutagenesis based on molecular docking analysis. Twelve mutants were generated and characterized in detail. Kinetic studies showed that the *K*_*m*_ values of the P160A, P160T, P160H, N161E, and D162W mutants were decreased, whereas *K*_*m*_ values of H159Y, H159V, H159S, N161R, N161F, D162K, and D162M mutants were increased. In addition, all mutants displayed decreased catalytic efficiency except for the P160A mutant, whose *k*_*cat*_/*K*_*m*_ was increased by 33.2%. Moreover, by overexpressing the P160A mutant, the engineered strain T.G-PMS-P160A was able to produce 5.2 g/L fumarate. To further enhance fumarate production, the acid tolerance of T.G-PMS-P160A was improved by deleting *ade12*, a component of the purine nucleotide cycle, and the resulting strain T.G(△ade12)-PMS-P160A produced 9.2 g/L fumarate. The strategy generated in this study opens up new avenues for pathway optimization and efficient production of natural products.

## Introduction

Fumarate, one of the top 12 biomass building block compounds, is widely used in food, pharmaceutical, and chemical industries [1,2]. Currently, natural producers of fumarate, such as *Rhizopus oryzae* [3], are attracting more attention, but fumarate production is limited in these organisms by their morphology and production characteristics [4]. Given this deficiency, considerable interest has been turned toward engineering microorganisms for improved fumarate production.

The vitamin auxotroph yeast *Torulopsis glabrata* has been extensively used to produce pyruvate, α-ketoglutarate, malate, and fumarate [5–8]. Vitamins such as thiamine, biotin, nicotinic acid, and pyridoxine, can be used to regulate carbon flux between cell growth and organic acid production [6,9,10]. Under optimal vitamin concentrations, 94.3 g/L pyruvate was obtained from *T*. *glabrata*, which provides a large quantity of precursors for organic acid biosynthesis [5]. In addition, *T*. *glabrata* is more glucose-tolerant than other strains such as *Saccharomyces cerevisiae* and *Escherichia coli* [11,12]. Moreover, high acid tolerance is essential for achieving higher cell density and sustained metabolism [13,14]. Thus, *T*. *glabrata* is a promising alternative host for metabolic engineering to redirect carbon flux from pyruvate to fumarate.

We explored four metabolic pathways to produce fumarate involving reductive reactions of oxaloacetate in *S*. *cerevisiae* and *R*. *oryzae* [15–17], citrate oxidation via the TCA cycle in *S*. *cerevisiae* and *T*. *glabrata* [18,19], the noncyclic glyoxylate route in *Escherichia coli* [12], and the urea and the purine cycle in *T*. *glabrata* [8] (Table 1). Among these pathways, fumarate production via the reductive TCA cycle provides a maximum theoretical yield of 2 mol/mol glucose (or 1.48 g/g glucose) [4]. Considering this advantage, an exogenous metabolic pathway involving the reductive TCA cycle was successfully introduced in *S*. *cerevisiae*, but the final engineered *S*. *cerevisiae* strain only produced 3.18 g/L fumarate [15]. One of the rate-limiting factors for fumarate production is associated with the low catalytic efficiency of fumarase from *R*. *oryzae* (RoFUM), which catalyzes the dehydration of malate to fumarate [20].

**Table 1:** Comparison of fumarate production by natural and metabolically engineered microorganisms.

Strains	Fumarate* (g/L)	Yield*(g/g)	Productivity* (g/L^/^h)	References
**Natural producers**				
*R*. *formosa*	21.3	0.34	-	[21]
*R*. *arrhizus*	38.0	0.33	0.46	[22]
*R*. *oryzae* ZJU11	41.1	0.48	0.37	[23]
*R*. *oryzae* ME-UN-8	52.7	0.53	0.55	[3]
*R*. *oryzae* FM19	56.5	0.70	0.67	[24]
**Engineered strains**				
*E*. *coli* CWF812	28.2	0.39	0.45	[12]
*R*. *oryzae* ppc	25	0.78	0.26	[16]
*R*. *oryzae* FM19	49.4	0.56	0.55	[25]
*S*. *cerevisiae* FMME 006 *ΔFUM1*+↑*RoPYC*+↑*RoMDH*+↑*RoFUM1*	5.64	0.11	0.06	[4]
T.G(△ade12)-PMS-P160A	9.2	0.15	0.15	This study

Note: * The actual titer, yield, and productivity of fumarate.

**Fig 1:**
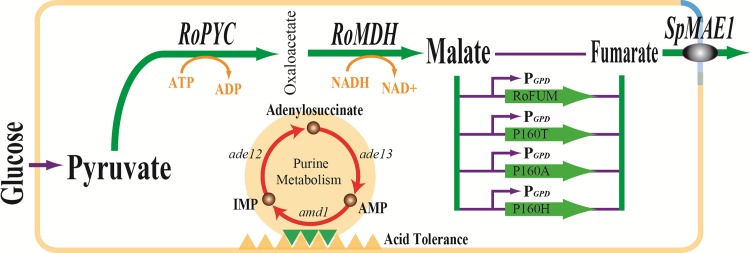
Major metabolic pathways leading to fumarate formation in *T*. *glabrata*. Boldface arrows indicate variants for fumarate synthesis implemented in strains featured in this study. RoPYC: pyruvate carboxylase from *R*. *oryzae*; RoMDH: malate dehydrogenase from *R*. *oryzae*; RoFUM: fumarase from *R*. *oryzae*; SpMAE1: C_4_-dicarboxylic acid transporter from *Schizosaccharomyces pombe*; ade12: adenylosuccinate synthase; ade13: adenylosuccinate lyase; amd1: AMP deaminase.

In this study, molecular docking was used to identify promising mutations in binding site B that might improve the catalytic efficiency of RoFUM. Then, proteins containing these promising mutations were expressed in *E*. *coli* BL21(DE3), and enzyme kinetic parameters were characterized to screen efficient RoFUM mutations. In addition, the purine nucleotide cycle was engineered to enhance acid tolerance. The final engineered strain, T.G(△ade12)-PMS-P160A, was able to produce 9.2 g/L fumarate (Fig 1).

## Materials and Methods

### Strains and plasmids

The multi-vitamin auxotrophic *T*. *glabrata* CCTCC M202019Δ*ura3*Δ*arg8* strain was used as the host strain for gene overexpression [26]. The engineered *T*. *glabrata* strain T.G-PMS was screened for malate production, in which pyruvate carboxylase (RoPYC) from *R*. *oryzae*, malate dehydrogenase (RoMDH) from *R*. *oryzae*, and C_4_-dicarboxylic acid transporter (SpMAE1) from *Schizosaccharomyces pombe* were simultaneously overexpressed [7]. The engineered yeast strains used for fumarate production in this study were derived from T.G-PMS (Table 2). Plasmids pY2X-SpMAE1 and pETDuet-1 were used for plasmid construction (Table 2).

**Table 2:** Strains and plasmids used in this study.

Strains and plasmids	Relevant characteristics	References
**Strains**		
*E*. *coli* BL21(DE3)	F^-^ *ompT hsdS*_*B*_ (*r*_*B*_^*-*^ *m*_*B*_^*-*^) *gal dcm* (DE3)	Novagen
*E*. *coli* BL21-RoFUM	*E*. *coli* BL21(DE3) (pETDuet-RoFUM)	This study
*E*. *coli* BL21-H159S	*E*. *coli* BL21(DE3) (pETDuet-H159S)	This study
*E*. *coli* BL21-H159Y	*E*. *coli* BL21(DE3) (pETDuet-H159Y)	This study
*E*. *coli* BL21-H159V	*E*. *coli* BL21(DE3) (pETDuet-H159V)	This study
*E*. *coli* BL21-P160A	*E*. *coli* BL21(DE3) (pETDuet-P160A)	This study
*E*. *coli* BL21-P160H	*E*. *coli* BL21(DE3) (pETDuet-P160H)	This study
*E*. *coli* BL21-P160T	*E*. *coli* BL21(DE3) (pETDuet-P160T)	This study
*E*. *coli* BL21-N161R	*E*. *coli* BL21(DE3) (pETDuet-N161R)	This study
*E*. *coli* BL21-N161E	*E*. *coli* BL21(DE3) (pETDuet-N161E)	This study
*E*. *coli* BL21-N161F	*E*. *coli* BL21(DE3) (pETDuet-N161F)	This study
*E*. *coli* BL21-D162W	*E*. *coli* BL21(DE3) (pETDuet-D162W)	This study
*E*. *coli* BL21-D162K	*E*. *coli* BL21(DE3) (pETDuet-D162K)	This study
*E*. *coli* BL21-D162M	*E*. *coli* BL21(DE3) (pETDuet-D162M)	This study
T.G*Δura3Δarg8*	CCTCC M202019*Δura3Δarg8*	[26]
T.G-PMS	CCTCC M202019*Δura3Δarg8* (pY26-RoPYC-RoMDH, pY2X-SpMAE1)	[7]
T.G-PMS-RoFUM	CCTCC M202019*Δura3Δarg8* (pY26-RoPYC-RoMDH, pY2X-SpMAE1-RoFUM)	This study
T.G-PMS-P160A	CCTCC M202019*Δura3Δarg8* (pY26-RoPYC-RoMDH, pY2X-SpMAE1-P160A)	This study
T.G(Δamd1)-PMS-P160A	CCTCC M202019*Δura3Δarg8Δamd1* (pY26-RoPYC-RoMDH, pY2X-SpMAE1-P160A)	This study
T.G(Δade12)-PMS-P160A	CCTCC M202019*Δura3Δarg8Δade12* (pY26-RoPYC-RoMDH, pY2X-SpMAE1-P160A)	This study
T.G(Δade13)-PMS-P160A	CCTCC M202019*Δura3Δarg8Δade13* (pY26-RoPYC-RoMDH, pY2X-SpMAE1-P160A)	This study
**Plasmids**		
pETDuet-1	ColE1, *Amp*, P_T7_, P_T7_	This study
pETDuet-RoFUM	ColE1, *Amp*, P_T7_-*RoFUM*, P_T7_	This study
pETDuet-H159S	ColE1, *Amp*, P_T7_-*H159S*, P_T7_	This study
pETDuet-H159Y	ColE1, *Amp*, P_T7_-*H159Y*, P_T7_	This study
pETDuet-H159V	ColE1, *Amp*, P_T7_-*H159V*, P_T7_	This study
pETDuet-P160A	ColE1, *Amp*, P_T7_-*P160A*, P_T7_	This study
pETDuet-P160H	ColE1, *Amp*, P_T7_-*P160H*, P_T7_	This study
pETDuet-P160T	ColE1, *Amp*, P_T7_-*P160T*, P_T7_	This study
pETDuet-N161R	ColE1, *Amp*, P_T7_-*N161R*, P_T7_	This study
pETDuet-N161E	ColE1, *Amp*, P_T7_-*N161E*, P_T7_	This study
pETDuet-N161F	ColE1, *Amp*, P_T7_-*N161F*, P_T7_	This study
pETDuet-D162W	ColE1, *Amp*, P_T7_-*D162W*, P_T7_	This study
pETDuet-D162K	ColE1, *Amp*, P_T7_-*D162K*, P_T7_	This study
pETDuet-D162M	ColE1, *Amp*, P_T7_-*D162M*, P_T7_	This study
pY2X-SpMAE1	2 μm, *Amp*, *ARG8*, P_GPD_, P_TEF_-*SpMAE1*	[7]
pY26-RoPYC-RoMDH	2 μm, *Amp*, *URA3*, P_GPD_-*RoMDH*, P_TEF_-*RoPYC*	[7]
pY2X-SpMAE1-RoFUM	2 μm, *Amp*, *ARG8*, P_GPD_-*RoFUM*, P_TEF_-*SpMAE1*	This study
pY2X-SpMAE1-P160A	2 μm, *Amp*, *ARG8*, P_GPD_-*P160A*, P_TEF_-*SpMAE1*	This study

### Expression of the *RoFUM* gene in *E*. *coli* BL21(DE3)

The *c*DNA of *R*. *oryzae* NRRL1526 (ATCC 10260) was used as a template to amplify the fumarase gene *RoFUM* (GenBank: HM130701) by PCR, which has previously been overexpressed in *S*. *cerevisiae* to enhance fumarate production [15]. The resulting PCR product was subcloned into pETDuet-1, and the identity of the final plasmid, pETDuet-RoFUM, was confirmed by sequencing (Sangon Biotech, Shanghai) and electrophoretic analysis (S1 Fig). The resulting pETDuet-RoFUM was transformed into *E*. *coli* BL21(DE3) (S2 Fig).

### Site-directed mutagenesis

The recombinant plasmid was amplified with mutagenic oligonucleotides according to the protocol accompanying the MutanBEST kit (TaKaRa, Dalian, China). After purification, the resulting fragments were blunted as described by the Blunting Kination Enzyme Mix (TaKaRa, Dalian, China). Then, these blunt-end fragments were ligated into pETDuet-1, and transformed into *E*. *coli* JM109 competent cells. Next, the presence of *RoFUM* and its mutants in the transformants was confirmed by PCR and DNA sequencing. Finally, recombinant plasmids were transformed into *E*. *coli* BL21(DE3) competent cells for expression (Table 2).

### Plasmids and strains construction

The mutant strain *T*. *glabrata* CCTCC M202019Δ*ura3*Δ*arg8*Δ*amd1* was obtained by genomic integration [27]. PCR products of the marker gene *loxP*-*kanMX*-*loxP* were amplified from the vector pUG27 [28], and the 5' and 3' regions flanking *amd1* (CAGL0G07425g, AMP deaminase) were amplified from the *T*. *glabrata* genome. Then, the flanking PCR product was generated by fusion PCR. After *T*. *glabrata* CCTCC M202019Δ*ura3*Δ*arg8* were transformed, the yeast strains were plated onto solid medium A (S3 Fig). Then, the fusion fragments were integrated into the genome via homologous recombination, and this result was verified by DNA sequencing and genomic PCR (S4 Fig). The mutant strains *T*. *glabrata* CCTCC M202019Δ*ura3*Δ*arg8*Δ*ade12* and *T*. *glabrata* CCTCC M202019Δ*ura3*Δ*arg8*Δ*ade13* were constructed by deleting *ade12* (CAGL0K05027g, adenylosuccinate synthase) and *ade13* (CAGL0B02794g, adenylosuccinate lyase), respectively, in the same manner as was done for *T*. *glabrata* CCTCC M202019Δ*ura3*Δ*arg8*Δ*amd1*.

Restriction endonucleases (Takara) and the DNA Ligase Kit Ver. 2.0 (Takara) were used to construct plasmids. In this manner, plasmids pY2X-SpMAE1-RoFUM and pY2X-SpMAE1-P160A were constructed. Then, plasmids were transformed into *T*. *glabrata* as previously described [26]. After this, the yeast strains were plated onto solid medium A. Plasmids pY26-RoPYC-RoMDH and pY2X-SpMAE1-RoFUM were simultaneously transformed into *T*. *glabrata* CCTCC M202019Δ*ura3*Δ*arg8* to yield the T.G-PMS-RoFUM strain. Plasmids pY26-RoPYC-RoMDH and T.G-PMS-P160A were simultaneously transformed into *T*. *glabrata* CCTCC M202019Δ*ura3*Δ*arg8*, *T*. *glabrata* CCTCC M202019Δ*ura3*Δ*arg8*Δ*amd1*, *T*. *glabrata* CCTCC M202019Δ*ura3*Δ*arg8*Δ*ade12*, and *T*. *glabrata* CCTCC M202019Δ*ura3*Δ*arg8*Δ*ade13*, resulting in strains T.G-PMS-P160A, T.G(Δamd1)-PMS-P160A, T.G(Δade12)-PMS-P160A and T.G(Δade13)-PMS-P160A, respectively.

### Media

Media were prepared as follows. Luria-Bertani (LB) medium: 10 g/L peptone, 5 g/L yeast extract, and 10 g/L NaCl, pH 7.0; medium A: 20 g/L glucose, 7 g/L NH_4_Cl, 5 g/L KH_2_PO_4_, 0.8 g/L MgSO_4_·7H_2_O, 3 g/L sodium acetate, 32 mg/L thiamine-HCl, 80 mg/L biotin, 0.8 mg/L pyridoxine-HCl, and 16 mg/L nicotinic acid; medium B: 60 g/L glucose, 7 g/L NH_4_Cl, 5 g/L KH_2_PO_4_, 0.8 g/L MgSO_4_·7H2O, 6.6 g/L K_2_SO_4_, 3 g/L sodium acetate, 12 mg/L thiamine-HCl, 30 mg/L biotin, 0.4 mg/L pyridoxine-HCl, and 8 mg/L nicotinic acid. After filter sterilization, all vitamins were added to the medium. After dry-heating sterilization at 160°C for 30 min, CaCO_3_ was used as a pH buffer.

### Culture conditions

LB medium was used for culturing *E*. *coli* bacteria and for producing fumarase on a rotary shaker at 200 rpm at 37°C. When *RoFUM* and its mutants were expressed in *E*. *coli* BL21(DE3), the inducer isopropyl β-D-1-thiogalactopyranoside (IPTG) was added to a final concentration of 0.4 mM when the optical density at 600 nm (OD_600_) reached 0.5. Then, cells were further cultured at 37°C for 6 h. *T*. *glabrata* strains were cultured as previously described [7]. The seed culture inoculated from a slant was cultivated for 24 h on a reciprocal shaker at 30°C, at 200 rpm, in a 25 mL/250 mL flask containing medium A. The broth was centrifuged, the supernatant liquid was removed and discarded, and the pellet was suspended in fresh medium B. Then, the cell suspension was divided equally between 500 mL flasks containing 50 mL fresh medium B with an initial biomass dry weight of 1 g/L for fermentation. The medium was buffered by the addition of 60 g/L CaCO_3_ followed by fermentation at 30°C for 60 h with shaking at 200 rpm.

### Purification of fumarase

*E*. *coli* BL21(DE3) cells harboring recombinant plasmids were cultured at 37°C in LB medium with the addition of 100 μg/mL ampicillin until the OD_600_ reached 1.0. Then, expression was induced with 0.4 mM IPTG for 4–6 h. Protein purification was performed as previously described [29,30].

### Enzyme assays

The fumarase activity produced using malate as the substrate was determined by measuring malate consumption at 250 nm [15]. The protein concentration was measured with the Lowry method [31].

### Docking

The structures of RoFUM and its mutants were obtained based on the known structure of fumarase (PDB ID: 3e04) using the Swiss Model server. Molecular docking was performed by the Autodock-based docking tool, and after 25 runs, binding energies were calculated with Yasara [32]. The overall fold of the model was energy-minimized using the Yamber3 force field [33]. Molecular docking was performed by the following strategy [32]: i) preparing the ligand and receptor for AutoGrid, such as adding the polar hydrogens and partial charges and defining the flexible residues; ii) preparing the grid parameter file, such as electrostatic potential maps and desolvation energy maps; iii) running AutoDock according to the ligand and receptor PDBQT files, grid maps, and docking parameter file; and iv) analyzing AutoDock results using AutoDockTools and creating images with PyMOL v.0.99. In this process, temperature and pH were not considered.

### Effect of temperature and pH on enzyme activity

The optimal pH of RoFUM and its mutants was assayed at 30°C by measuring its activity across a pH range of 5.1–9.3 using citric acid/sodium phosphate buffer (pH 5.1–7.1) and sodium carbonate/sodium bicarbonate buffer (pH 8.0–9.4). Relative enzyme activities were determined and the activity of the enzyme without incubation was defined as 100%. The optimal temperature of RoFUM and its mutant was measured between 15–35°C in 20 mM phosphate buffer at pH 7.3 using malate as the substrate. At each temperature, the buffer and malate were preincubated for 10 min. Then, the enzyme was added to initiate the reaction, and the reaction was allowed to proceed for 30 min. Finally, the reaction was heated at 100°C for 10 min to inactivate the enzyme. All values presented in graphs are the means of three replications.

### Influence of mutations on kinetic parameters

The kinetic parameters (*K*_*m*_, *k*_*cat*_, and *k*_*cat*_*/K*_*m*_) of RoFUM and its mutants were measured in phosphate buffer (pH 7.3, 20 mM) at 30°C. Assays were performed with enzyme and substrates of different concentrations from 0.15–15 mM. *K*_*m*_ was estimated from Eadie-Hofstee plots [34]. All values presented are the means of three replications.

### Analytical methods

The optical absorbance at 660 nm (OD_660_) was converted to dry cell weight (DCW) according to a predetermined calibration equation [35]:

OD660/DCW=1/0.23g/L

Glucose, pyruvate, malate, and fumarate levels were determined using high-performance liquid chromatography (HPLC). Glucose was detected by Agilent 1200 with refractive index detector, using an Aminex HPX-87H column eluted with 5 mM H_2_SO_4_ at a flow rate of 0.6 mL/min at 35°C [15]. Pyruvate, malate, and fumarate were determined by HPLC using a Dionex Acclaim 120 C_18_ reversed-phase analytical column at a flow rate of 0.6 mL/min [7].

### Tolerance assays

The cell growth of *T*. *glabrata* was measured by spotting 5 μL of 10-fold dilutions of logarithmic-phase yeast onto solid medium B under different pH conditions [36]. After incubation at 30°C for 4 days, colonies were visualized on the plates.

### Statistical analysis

All measurements were taken in triplicate and experiments were repeated three times to calculate the standard deviation. These values were used to compute *P* values using SPSS 16.0 (SPSS). Statistical significance of differences was evaluated with the Student’s *t* test.

## Results

### Selecting mutation sites based on docking analysis

**Fig 2:**
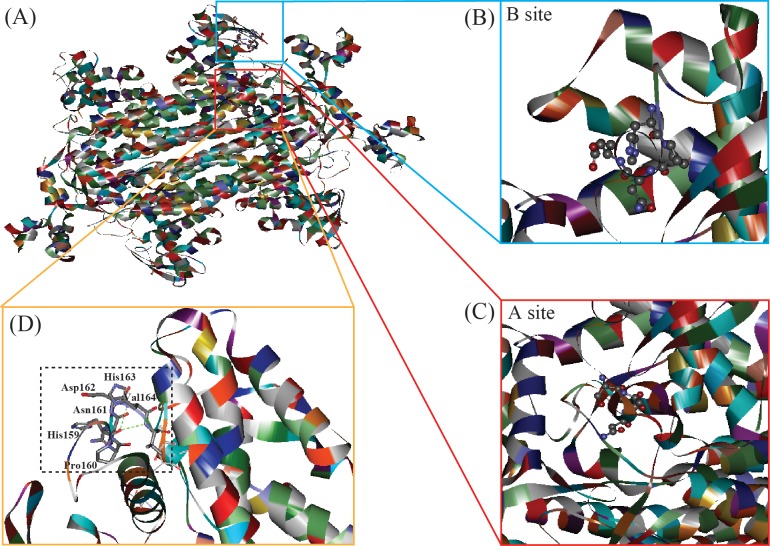
Structural model of RoFUM constructed by the Swiss Model server. The positions of the residues that are critical for substrate binding in RoFUM are shown as “sticks”. (A) Overview, (B) binding site B, (C) binding site A, and (D) the residues in binding site A.

To determine the mutation sites of fumarase, a 3D model of RoFUM was constructed based on the known structure of fumarase (PDB ID: 3e04) using the Swiss Model server (Fig 2A). The amino acid sequence of RoFUM includes two substrate-binding sites that were previously identified in the fumarase subfamily (Fig 2B and 2C) [37–39]: ^130^T^169^SSN^171^ (A site) and ^159^HPND^162^ (B site). The A site is the catalytic site, and the B site may play a role in the transfer of substrate or product between the active site and the solvent.

To engineer the catalytic efficiency of RoFUM, docking studies were carried out using the fumarase model as a receptor and malate and fumarate as ligands to investigate enzyme-substrate interactions. In addition, amino acid replacements in the B site were introduced into the RoFUM homology model, and the resulting variants were energy-minimized and docked with malate and fumarate. The binding energies of the H159S, H159Y, H159V, P160A, P160H, P160T, N161R, N161E, N161F, D162W, D162K, and D162M mutants to malate were lower than that observed for the models docked to fumarate (Table 3), indicating that these mutants might exhibit improved enzymatic properties compared to wild-type RoFUM.

**Table 3:** Docking energy of fumarase.

Mutation	Fumarate (Kcal/mol)	Malate (Kcal/mol)	Mutation	Fumarate (Kcal/mol)	Malate (Kcal/mol)	Mutation	Fumarate (Kcal/mol)	Malate (Kcal/mol)	Mutation	Fumarate (Kcal/mol)	Malate (Kcal/mol)
**RoFUM**	**-4.81**	**-3.95**									
H159A	-4.50	-4.80	**P160A**	**-1.41**	**-3.53**	N161A	-4.97	-4.08	D162C	-4.74	-4.25
H159C	-4.54	-3.85	P160C	-4.45	-2.43	N161C	-4.08	-2.44	D162E	-4.69	-4.19
H159D	-4.73	-4.28	P160D	-4.52	-3.87	**N161E**	**-3.05**	**-4.40**	D162F	-4.74	-4.31
H159E	-4.49	-4.81	P160E	-4.58	-2.58	**N161F**	**-4.56**	**-5.02**	D162H	-4.82	-2.85
H159F	-4.75	-4.35	P160F	-4.86	-4.41	N161G	-4.21	-2.45	D162I	-4.97	-3.45
H159G	-4.58	-4.48	P160G	-1.10	-0.20	N161H	-4.98	-3.54	**D162K**	**-4.80**	**-5.03**
H159I	-4.65	-3.94	**P160H**	**-4.60**	**-5.49**	N161I	-4.04	-2.33	D162L	-4.92	-2.90
H159K	-4.63	-4.39	P160I	-4.21	-2.44	N161K	-4.65	-4.57	**D162M**	**-4.82**	**-5.01**
H159L	-4.58	-4.71	P160K	-4.49	-2.44	N161L	-4.63	-4.24	D162N	-4.88	-4.18
H159M	-4.65	-4.22	P160L	-4.11	-2.44	N161M	-4.69	-4.65	D162Q	-4.96	-5.13
H159N	-4.58	-4.67	P160M	-4.42	-3.33	N161P	-4.97	-4.95	D162R	-4.87	-3.52
H159P	-4.73	-4.98	P160N	-4.46	-2.52	N161Q	-4.61	-4.10	D162T	-4.06	-2.97
H159Q	-4.69	-4.86	P160Q	-4.44	-2.52	**N161R**	**-4.57**	**-4.94**	D162V	-4.74	-2.99
H159R	-4.65	-4.03	P161R	-1.12	-0.74	N161T	-4.66	-4.66	**D162W**	**-4.79**	**-5.11**
**H159S**	**-4.68**	**-5.17**	P160S	-4.51	-2.60	N161V	-4.45	-4.54	D162Y	-5.03	-5.05
**H159V**	**-4.56**	**-4.89**	**P160T**	**-4.03**	**-4.81**	N161W	-4.52	-2.33			
H159W	-4.89	-4.86	P160Y	-4.04	-2.48	N161Y	-4.54	-2.90			
**H159Y**	**-4.57**	**-4.96**									

Note: Bold mutations were selected for further study.

### Expression of mutant proteins

**Fig 3:**
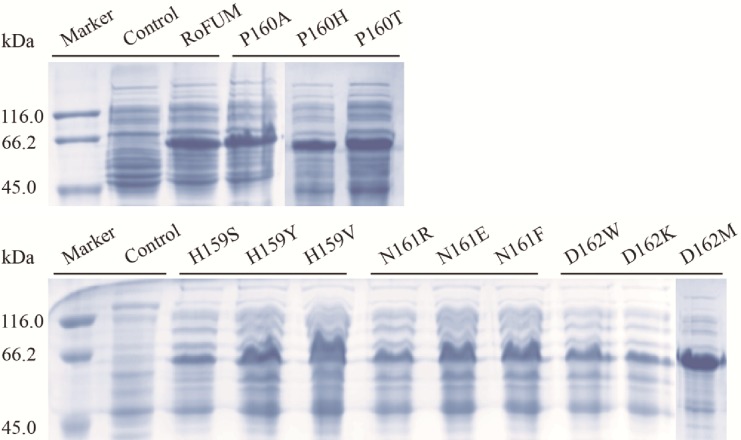
SDS-PAGE analysis of wild-type RoFUM and mutant proteins.

Based on the above analysis, H159S, H159Y, H159V, P160A, P160H, P160T, N161R, N161E, N161F, D162W, D162K, and D162M mutants were constructed by site-directed mutagenesis. After confirming the gene sequence, the mutant genes were cloned into pETDuet-1 and expressed in *E*. *coli* BL21(DE3). SDS-PAGE analysis indicated that the proteins were approximately 60 kDa (Fig 3), as expected.

### Effects of temperature and pH on enzyme activity

**Fig 4:**
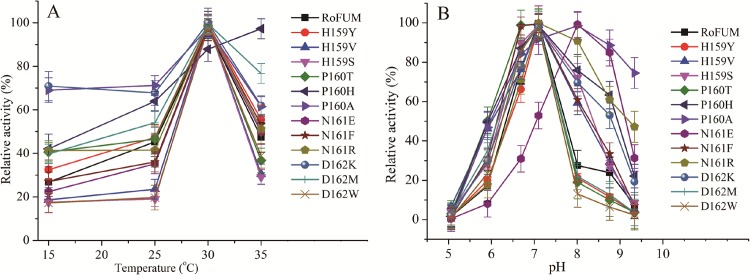
Effect of temperature and pH on RoFUM and its mutants. (A) The effect of temperature on the activity of RoFUM and its mutants was measured at pH 7.3 with temperatures ranging from 15–35°C. (B) The effect of pH on RoFUM (quadrangle, black), H159Y (circle, red), H159V (upper triangle), H159S (reverse triangle), P160T (quadrangle, green), P160H (left triangle), P160A (right triangle), N161E (sexangle), N161F (star), N161R (pentacle), D162K (circle, blue), D162M (vertical bar), D162W (cross). All values presented in graphs are the means of three replications.

Malate production is usually performed at 30°C and pH 6.0–7.0 in a 5-L fermentor by the engineered *T*. *glabrata* T.G-PMS (data not shown) [7] and *S*. *cerevisiae* RWB525 [40], under which PYC and MDH exhibit their maximum activity. In this study, we planned to construct the pathway for fumarate production in T.G-PMS by overexpressing RoFUM or its mutants (Fig 1). In order to act in conjunction with RoPYC and RoMDH, RoFUM or its mutants should display high activity under these conditions. Enzyme activities were determined from 15–35°C using malate as the substrate. The optimal temperature was 30°C for all mutants, which was similar to the optimal temperature of wild-type RoFUM (Fig 4A). Then, the activities of RoFUM and its mutants were measured at various pH values. The optimal pH of H159S, H159Y, H159V, P160H, N161R, D162W, D162K, and D162M mutants was 7.1, similar to wild-type RoFUM (Fig 4B), and the P160A, P160T, N161E, and N161F mutants displayed a slight shift in optimal pH (Fig 4B). These results demonstrated that RoFUM and its mutants were suitable for producing fumarate at 30°C and pH 7.0.

### Influence of mutations on kinetic parameters

One of the rate-limiting factors for fumarate production is the low catalytic efficiency of RoFUM [15]. To circumvent this potential bottleneck in fumarate production, mutant fumarases were screened by kinetic analysis. The kinetic parameters of RoFUM and its mutants were analyzed at 30°C using malate as the substrate (Table 4). Compared with RoFUM, the Michaelis constant (*K*_*m*_) values of P160A, P160T, P160H, N161E, and D162W mutants were decreased by 53.2%, 39.0%, 2.6%, 72.7%, and 62.3%, respectively, whereas *K*_*m*_ values of H159Y, H159V, H159S, N161R, N161F, D162K, and D162M mutants were increased by 123.4%, 120.8%, 36.4%, 39.0%, 58.4%, 89.6%, and 45.5%, respectively. In addition, the catalytic constants (*k*_*cat*_) of all the mutants were reduced except for the D162K mutant, whose *k*_*cat*_ was increased by 17.3%, and the *k*_*cat*_ of the P160A mutant was decreased by 37.4%. Moreover, compared to wild-type RoFUM, all mutants showed a large decrease in the catalytic efficiency (*k*_*cat*_/*K*_*m*_) except for the P160A mutant whose *k*_*cat*_/*K*_*m*_ was increased by 33.2%. These results indicated that malate could be more effectively converted to fumarate by the P160A mutant compared to wild-type RoFUM and other mutants, suggesting that further stepwise improvement could be made in fumarate production by overexpressing P160A in the *T*. *glabrata* strain T.G-PMS.

**Table 4:** Effect of site-directed mutagenesis on the kinetic parameters of fumarase.

Mutations	*K*_m_ (×10^-2^mM)	*k*_cat_ (×10^−2^/min)	*k*_cat_/*K*_m_ (/min·mM)	*k*_cat_/*K*_m_ Change (%) (B/A-1) × 100
**RoFUM**	57.4 ± 0.8	333.1 ± 12.5	5.8 ± 0.7	-
**H159Y**	128.2 ± 1.3	588.6 ± 13.7	4.5 ± 0.4	-22.41*
**H159V**	126.7 ± 2.4	287.6 ± 9.8	2.2 ± 0.3	-62.07**
**H159S**	78.3 ± 0.4	239.6 ± 10.2	3.0 ± 0.8	-48.28*
**P160T**	35.0 ± 0.1	170.7 ± 5.4	4.8 ± 0.6	-17.24*
**P160A**	26.8 ± 0.3	208.4 ± 9.0	7.7 ± 0.7	32.76*
**P160H**	55.9 ± 0.7	198.0 ± 0.1	3.5 ± 0.1	-39.66*
**N161R**	79.7 ± 2.1	268.0 ± 11.0	3.3 ± 0.5	-43.10*
**N161E**	15.6 ± 0.1	40.8 ± 5.4	2.6 ± 0.5	-55.17*
**N161F**	90.9 ± 1.2	175.5 ± 6.4	1.9 ± 0.7	-67.24**
**D162K**	108.8 ± 0.6	390.7 ± 3.2	3.5 ± 0.7	-39.66*
**D162M**	83.5 ± 0.7	333.6 ± 15.5	3.9 ± 0.5	-32.76*
**D162W**	21.6 ± 0.0	64.9 ± 1.9	3.0 ± 0.4	-48.28*

*k*_cat_/*K*_m_ Change was computed relative to the wild-type RoFUM enzyme.

*P < 0.05

**P < 0.01.

### Effect of mutations on fumarate production

**Fig 5:**
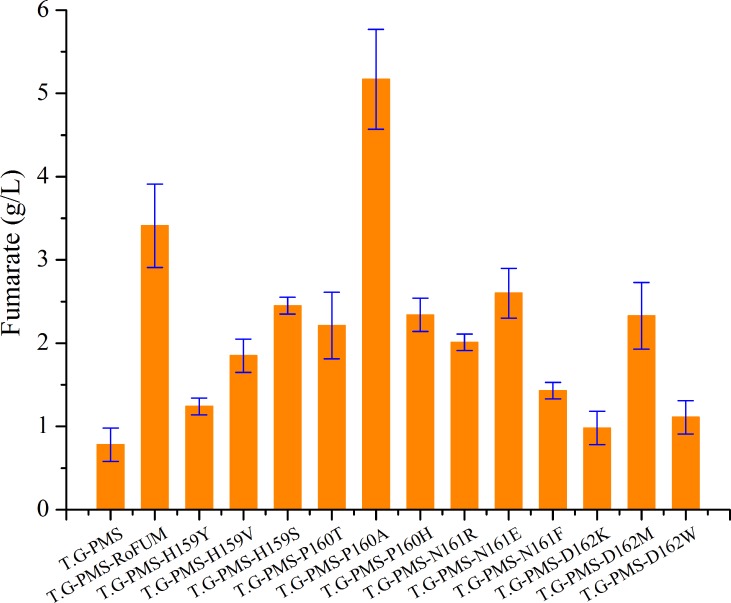
Effect of mutations on fumarate production.

**Fig 6:**
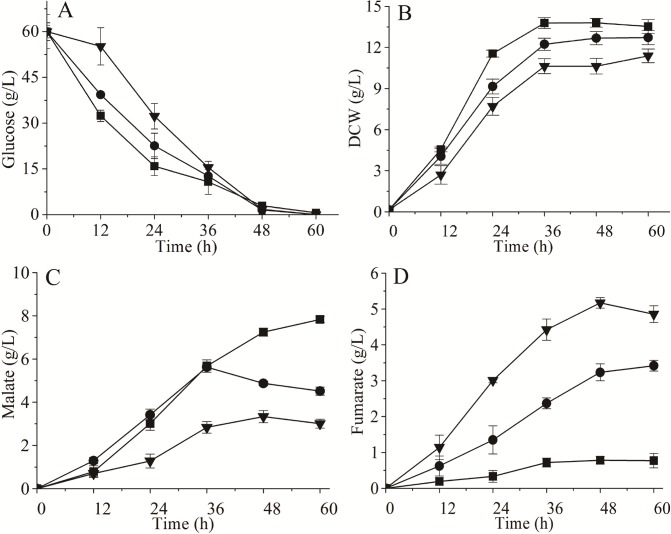
Effect of mutations on fermentation parameters. (A) Glucose consumption, (B) cell growth, (C) malate production, (D) fumarate production. ■T.G-PMS, ●T.G-PMS-RoFUM, ▼T.G-PMS-P160A. All values presented in graphs are the means of three replications.

Malate concentrations up to 8.5 g/L were obtained with strain T.G-PMS, in which RoPYC, RoMDH, and SpMAE1 were simultaneously overexpressed [7]. Thus, T.G-PMS can be exploited as a suitable and promising host for biotechnological production of fumarate through metabolic engineering. To investigate the effect of different RoFUM mutations on fumarate production, twelve mutants were overexpressed in the engineered strain T.G-PMS. All strains showed decreased fumarate production except for T.G-PMS-P160A, where fumarate production was increased compared to the T.G-PMS-RoFUM strain (Fig 5). These findings were consistent with kinetic studies on RoFUM and its mutants. To further study the effect of the P160A mutant on fumarate fermentation, fermentation parameters were determined, and the results indicated that overexpression of RoFUM and P160A in the engineered strain T.G-PMS could channel more malate flux toward fumarate production. When RoFUM and P160A were overexpressed in T.G-PMS, malate production was decreased by 28.2% and 57.4% compared to that of the control strain T.G-PMS, respectively (Fig 6C). These dramatic results corresponded to a 3.4- and 5.6-fold increase in fumarate titer compared to that of the control strain T.G-PMS, respectively (Fig 6D). Furthermore, compared to the engineered strain T.G-PMS-RoFUM, the engineered strain T.G-PMS-P160A exhibited a 51.2% increase in fumarate production to 5.2 g/L (Fig 6D), and the percentage of the actual and maximum theoretical yield of fumarate was increased to 5.8%. In addition, there was a 57.5% decrease in malate production to 3.3 g/L (Fig 6C). These results indicated that the P160A mutant allowed fumarate to be generated out of the malate node in the enhanced reductive TCA pathway.

### Engineering acid tolerance to enhance fumarate production

**Fig 7:**
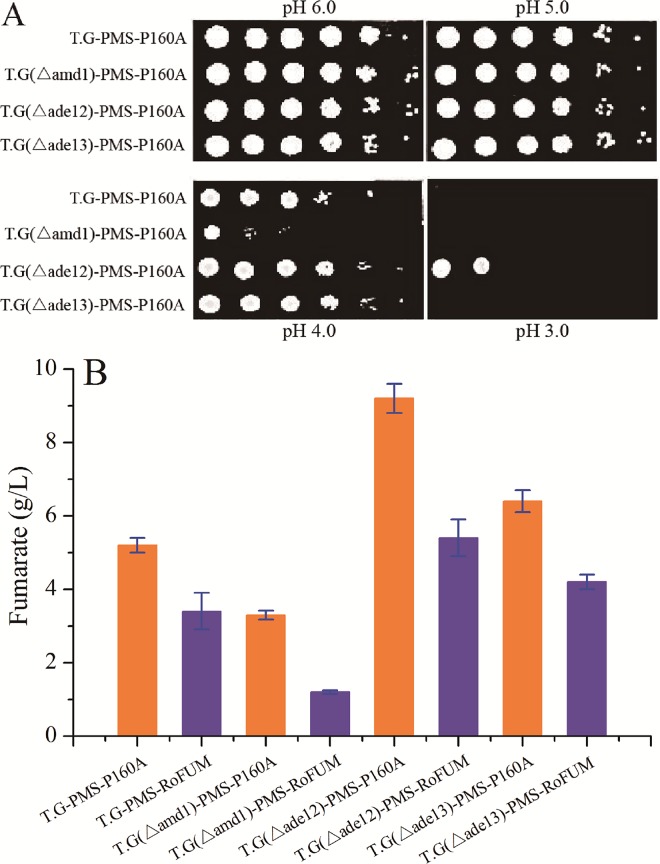
Effect of gene deletions on acid tolerance and fumarate production. (A) Growth assays under various pH values. Logarithmic-phase cells of each *T*. *glabrata* strain were adjusted to 2×10^7^ cells/mL, and then 5 μL of serial 10-fold dilutions were spotted onto the corresponding fermentation medium. Pictures were taken after 4 days of growth at 30°C. (B) Concentrations of fumarate obtained in shake flask cultivation of the different strains. All values presented in graphs are the means of three replications.

During organic acid production by *T*. *glabrata*, the pH in the culture broth gradually decreases as organic acids accumulate. As a result, glucose consumption and cell growth reduce in the case of T.G-PMS-RoFUM and T.G-PMS-P160A (Fig 6A and 6B). A previous study showed that deleting *ade12* and *ade13* genes in *T*. *glabrata* leads increased organic acid tolerance [41]. Thus, we engineered the purine nucleotide cycle to enhance acid tolerance by deleting *amd1*, *ade12*, and *ade13*, respectively. Growth was examined on fermentation medium over a pH range from 6.0 to 3.0. T.G(△amd1)-PMS-P160A, T.G(△ade12)-PMS-P160A, and T.G(△ade13)-PMS-P160A exhibited the same growth pattern as T.G-PMS-P160A in pH 6.0 to 5.0 (Fig 7A). The growth of T.G(△amd1)-PMS-P160A was severely diminished at pH values below 4.0, but T.G(△ade12)-PMS-P160A and T.G(△ade13)-PMS-P160A showed a slight increase in resistance to pH 4.0 compared to T.G-PMS-P160A (Fig 7A). Furthermore, in pH values below 3.0, T.G(△ade12)-PMS-P160A exhibited resistance against acid stress (Fig 7A). These results indicated that the acid tolerance of T.G-PMS-P160A can be significantly improved by deleting *ade12*. Finally, higher fumarate titers up to 9.2 g/L and 5.1 g/L were obtained with strain T.G(△ade12)-PMS-P160A and T.G(△ade12)-PMS-RoFUM, which was increased by 76.9% and 50.0% compared to T.G-PMS-P160A and T.G-PMS-RoFUM, respectively (Fig 7B). In addition, the percentage of the actual and maximum theoretical yield of fumarate with T.G(△ade12)-PMS-P160A was increased to 10.3%.

## Discussion

In an attempt to resolve the bottleneck that occurs in fumarate production, fumarase was engineered for improved catalytic efficiency. Based on molecular docking, twelve mutants were generated by site-directed mutagenesis. Kinetic studies showed that the *K*_*m*_ of the P160A mutant was decreased by 53.2%, and its *k*_*cat*_/*K*_*m*_ was increased by 33.2%. In addition, when the P160A mutant was overexpressed in T.G-PMS, the fumarate titer increased to 5.2 g/L. Furthermore, when the purine nucleotide cycle was engineered to enhance acid tolerance, the highest fumarate titer, up to 9.2 g/L, was obtained with strain T.G(△ade12)-PMS-P160A. These results lay a good foundation for further study of fumarate production engineering strategies.

Recently, protein engineering has become important for modifying natural proteins and enzymes to meet the needs of different industrial applications by improving enzyme activity or catalytic efficiency, changing substrate or product specificity, enhancing enzyme stability, and modifying cofactor usage [42,43]. In this study, according to the promising mutations predicted by a computational model, twelve mutants were generated and characterized in detail, but only one RoFUM mutation, P160A, was effective in improving enzyme function. The reason may lie in the 3D model of RoFUM and its mutations, which were constructed based on the known structure of fumarase (PDB ID: 3e04) using the Swiss Model server. Although the amino acid sequence identity is 74%, there are many subtle differences between RoFUM and 3e04. In addition, the adverse impact on the kinetic parameters observed is possibly due to the complexity of protein structure and function [44]. Generally, based on optimizing wild-type protein structures, site-directed mutagenesis is beneficial for improving kinetic parameters [45]. A careful comparison of the P160A mutant and wild-type RoFUM showed that the P160A mutant exhibited greater substrate affinity than RoFUM, but the *k*_*cat*_ of the P160A mutant was lower than that of wild-type RoFUM (Table 4). This may be due to the fact that the flexibility of the substrate binding site B is reduced in the P160A mutant, while this change results in a significant decrease in its *k*_*cat*_ compared to the wild-type RoFUM. In other words, introducing alanine residues transforms the RoFUM catalytic site into a proper environment for malate, although due to the restricted volume of the binding pocket, side effects on substrate turnover are observed. These results suggest that in order to improve both turnover and binding, an iterative site-directed mutagenesis strategy [46] is required to readjust the aromatic nature of the malate binding pocket.

Furthermore, protein engineering is also an efficient way to improve *in vivo* performance and enhance pathway productivity by changing kinetic parameters of key enzymes in metabolic pathways [42]. In this study, fumarate production was enhanced by overexpressing the P160A mutant, and the final engineered strain, T.G-PMS-P160A, produced 5.2 g/L fumarate, which was about 51.2% greater than production by the control strain T.G-PMS-RoFUM. This result indicated that compared with wild-type RoFUM and the mutants examined in this study, the P160A mutant is most beneficial for developing an efficient substrate channel for fumarate production. Overexpressing the P160A mutant possibly results not only in a moderate increase in fumarate flux, but also in a balanced expression of multiple pathways [47]. In other words, it can simultaneously optimize entire biosynthesis pathways and metabolic networks, thus achieving optimal performance for each biological system [48,49]. Another successful example of this approach is polyhydroxyalkanoate (PHA) production [50]. In this example, PHA synthase from *Aeromonas punctata* was engineered, and five variants exhibited as much as a 5-fold improvement over the wild-type, thus leading to a 126% increase in PHA accumulation. Therefore, the strategy described in this study should be widely applicable for engineering microbial hosts to produce other valuable metabolites.

Synthetic biology has enabled the production of many chemicals from renewable resources, such as organic acids. However, when synthetic pathways are simply assembled from biological components, they may not function optimally in biological systems [51]. This is partly due to the fact that during organic acid production by industrial microorganisms, the pH of the culture broth gradually decreases along with acid accumulation, resulting in inhibited cell growth and acid production [52]. To further increase organic acid production, neutralizing agents such as NaOH, CaCO_3_, and Na_2_CO_3_ are added to the fermentation broth [53], but this does not solve the problem fundamentally. In this study, *amd1*, *ade12*, and *ade13* genes in the purine nucleotide cycle were respectively deleted, and acid tolerance of the T.G(△ade12)-PMS-P160A strain was highly elevated. Further, the strain T.G(△ade12)-PMS-P160A produced 9.2 g/L fumarate, which was increased by 76.9% compared to T.G-PMS-P160A. These results are probably due to the improved ATP supply, which helps to maintain higher pH gradients in the system [53]. Studies have indicated that pH gradients between the cytoplasm and vacuole hold an important position and play a significant role in the acid tolerance of eukaryotic cells [54,55]. However, this function is only performed in a certain normal range of cytoplasmic pH [56,57] or vacuolar pH [54,58,59] values. Thus, maintaining this range requires two groups of active transporters, plasma ATPases and vacuole H^+^-ATPases, which are powered by a large amount of ATP [54]. Given this, increasing the ATP supply by deleting *ade12* is an efficient strategy for improving growth performance and fumarate production under more acidic conditions, such as pH 4.0–3.0.

## Supporting Information

S1 Fig Electrophoretic analysis of the recombinant plasmids after cutting with restriction endonuclease. (TIF)

S2 Fig Confirmation of the positive *E*. *coli* BL21(DE3) transformants by electrophoretic analysis. (TIF)

S3 Fig Screening mutant *T*. *glabrata* strains on plates. Cgamd1△: *T*. *glabrata* CCTCC M202019Δ*ura3*Δ*arg8*Δ*amd1*, Cgade12△: *T*. *glabrata* CCTCC M202019Δ*ura3*Δ*arg8*Δ*ade12*, and Cgade13△: *T*. *glabrata* CCTCC M202019Δ*ura3*Δ*arg8*Δ*ade13*.
(TIF)

S4 Fig Confirmation of the mutant *T*. *glabrata* strains by electrophoretic analysis. (a) *T*. *glabrata* CCTCC M202019Δ*ura3*Δ*arg8*Δ*amd1*; (b) *T*. *glabrata* CCTCC M202019Δ*ura3*Δ*arg8*Δ*ade12*; (c) *T*. *glabrata* CCTCC M202019Δ*ura3*Δ*arg8*Δ*ade13*.
(TIF)
