# Supplementary figures and images for: Fumarate Production by Torulopsis glabrata: Engineering Heterologous Fumarase Expression and Improving Acid Tolerance
Source: PLoS One. 2016 Oct 6;11(10):e0164141. doi: 10.1371/journal.pone.0164141 (PMC5053504; doi:10.1371/journal.pone.0164141)

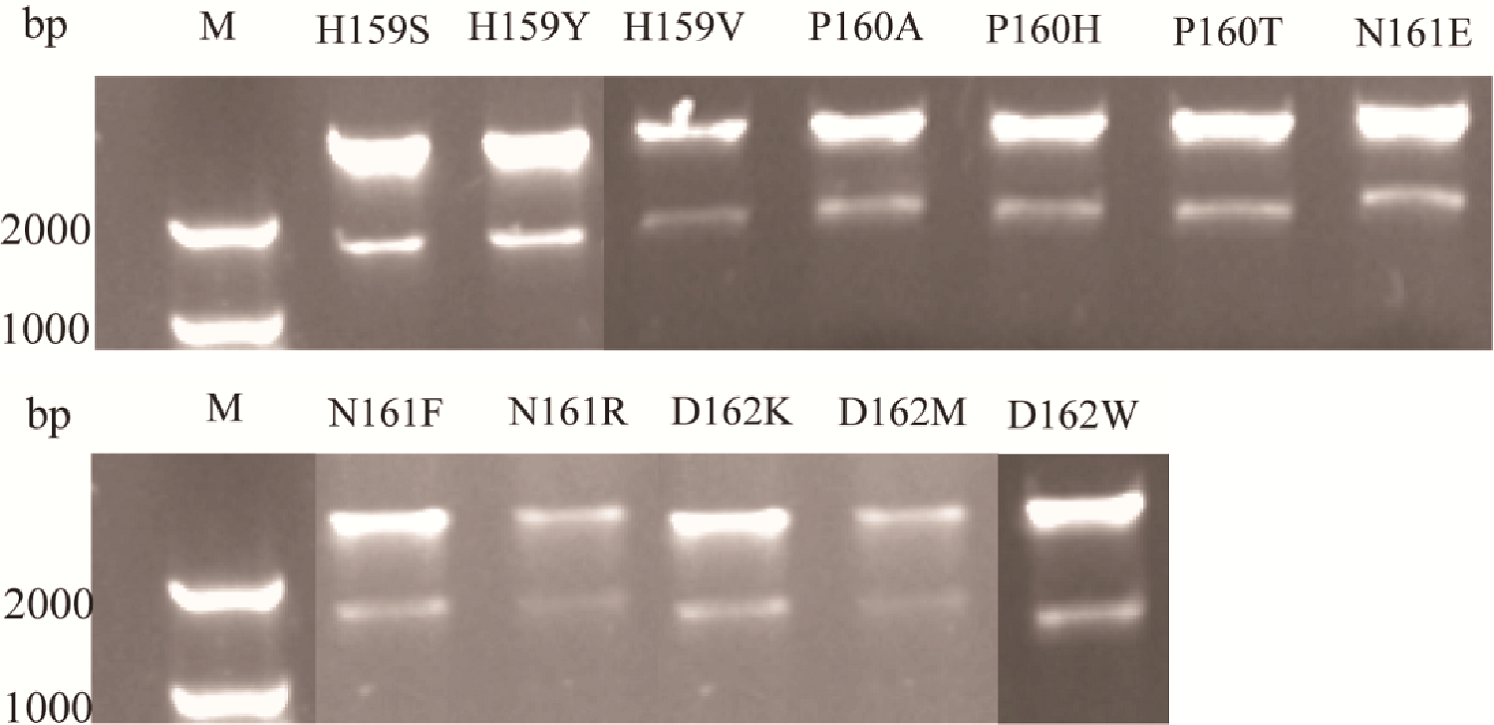

Supplement: S1 Fig — (TIF) [file pone.0164141.s001.tif]

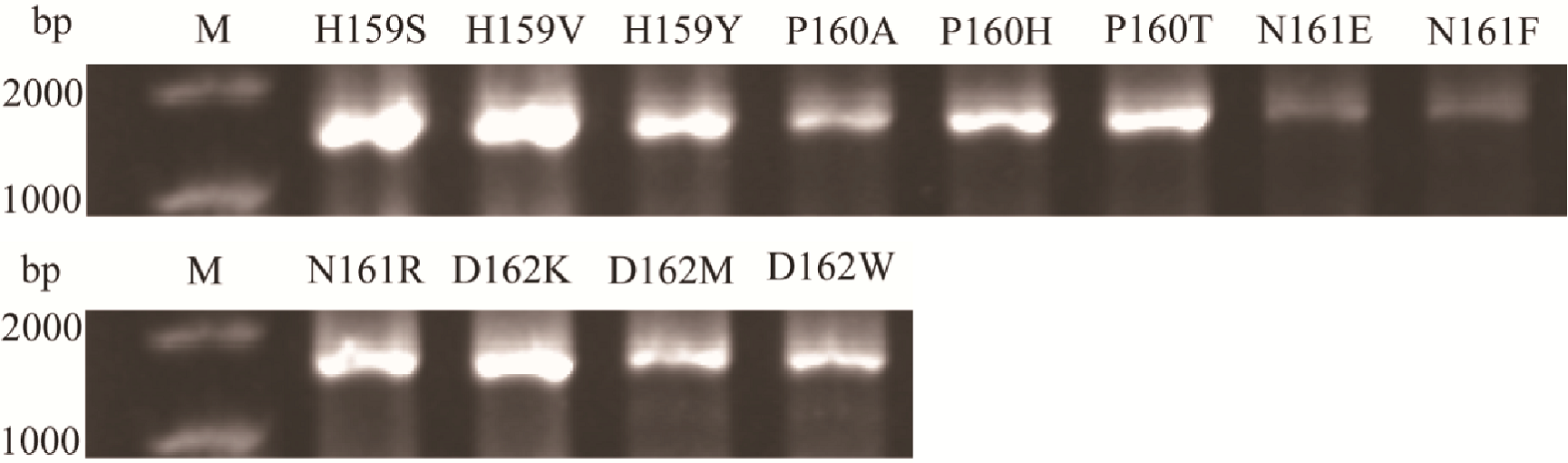

Supplement: S2 Fig — (TIF) [file pone.0164141.s002.tif]

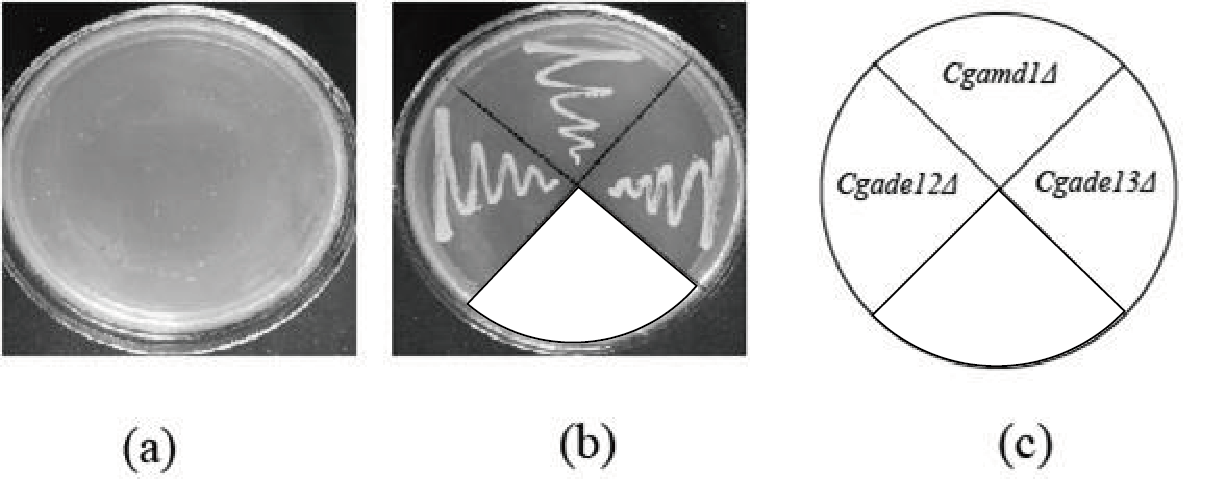

Supplement: S3 Fig — Cgamd1△: T. glabrata CCTCC M202019Δura3Δarg8Δamd1, Cgade12△: T. glabrata CCTCC M202019Δura3Δarg8Δade12, and Cgade13△: T. glabrata CCTCC M202019Δura3Δarg8Δade13. (TIF) [file pone.0164141.s003.tif]

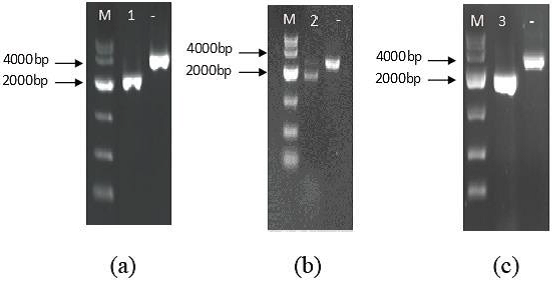

Supplement: S4 Fig — (a) T. glabrata CCTCC M202019Δura3Δarg8Δamd1; (b) T. glabrata CCTCC M202019Δura3Δarg8Δade12; (c) T. glabrata CCTCC M202019Δura3Δarg8Δade13. (TIF) [file pone.0164141.s004.tif]
